# The origin of the medial femoral circumflex artery, lateral femoral circumflex artery and obturator artery

**DOI:** 10.1007/s00276-018-2012-6

**Published:** 2018-04-12

**Authors:** M. Zlotorowicz, M. Czubak-Wrzosek, P. Wrzosek, J. Czubak

**Affiliations:** 1 Centre of Postgraduate Medical Education, Warsaw, Poland; 2 Department of Orthopaedics, Pediatric Orthopaedics and Traumatology, Gruca Teaching Hospital, Otwock, Poland

**Keywords:** Medial femoral circumflex artery, Lateral femoral circumflex artery, Obturator artery, Corona mortis, Femoral artery, Deep femoral artery

## Abstract

**Introduction:**

The most significant blood vessel supplying the hip joint is the medial femoral circumflex artery (MFCA). MFCA with lateral femoral circumflex artery (LFCA) are the first branches of the deep femoral artery (DFA) or they originate directly from the common femoral artery (CFA) or superficial femoral artery (SFA).

**Purpose and methods:**

We analyzed 100 CT angiogram of the hip region [72 men, 28 women; mean age 46.4 (14–80)] to assess the frequency of each type of division of the MFCA and LFCA from either the DFA or directly from the CFA or SFA. To assess the variations on each side in one individual we analyzed both hips in 73 patients [mean age 46.6 (14–80)].

**Results:**

Many different types of division have been described. The most frequent one in which both the MFCA and LFCA originate from the DFA, was observed in 50% of patients. In 31% of hips the MFCA originates from the CFA. In our study, a normal origin of the obturator artery from the internal iliac artery was observed in 67% of patients and an atypical origin, called corona mortis was observed in 33% of patients.

**Conclusions:**

The deep branch of the MFCA is the main artery supplying the femoral head, it is at risk during surgical approach to the hip joint. The atypical anastomosis called corona mortis is also at risk while performing the approach to pubic bone. Therefore, knowledge of their topography is very important.

## Introduction

The femoral artery is the main blood vessel of the lower limb. The significant branch of the femoral artery is the deep femoral artery, which is the main artery of the thigh providing the blood supply to the hip joint, femur, and muscles of the thigh.

The most influential blood vessel supplying the hip joint is the medial femoral circumflex artery (MFCA). The lateral femoral circumflex artery (LFCA) supplies the soft tissues around the hip joint. These two are the first branches of the deep femoral artery or they originate directly from the common femoral artery or superficial femoral artery. There are also two other vessels which can provide some blood supply to the femoral head: piriformis branch of the inferior gluteal artery and obturator artery via the foveal artery. The obturator artery has many anatomical variations. The most common one, called corona mortis has been a subject of many studies, although there are no studies describing corona mortis in the aspect of hip vascularity [7–9, 25, 26].

## Materials and methods

We analyzed 100 computed tomography angiograms of the hip region [72 men, 28 women; mean age, 46.4 (14–80); 50 right, 50 left hips—randomly selected from each individual] to assess the frequency of each type of division of the MFCA and LFCA from either the deep femoral artery or directly from the common or superficial femoral artery.

To assess the variations on each side in one individual we analyzed both hips in 73 patients [mean age, 46.6 (14–80) years; 53 men, 20 women].

We analyzed studies with good visualization of the small arteries in the hip region (middle-to-late arterial phase imaging) with intravenously administered contrast material. We excluded studies with sclerotic changes in the arteries visible in the angiographic images. The acquired data were evaluated with a combination of axial scans, multiplanar reformations and post-processing using a volume rendering technique and maximum intensity projection [5, 12, 25].

## Results

**Fig. 1:**
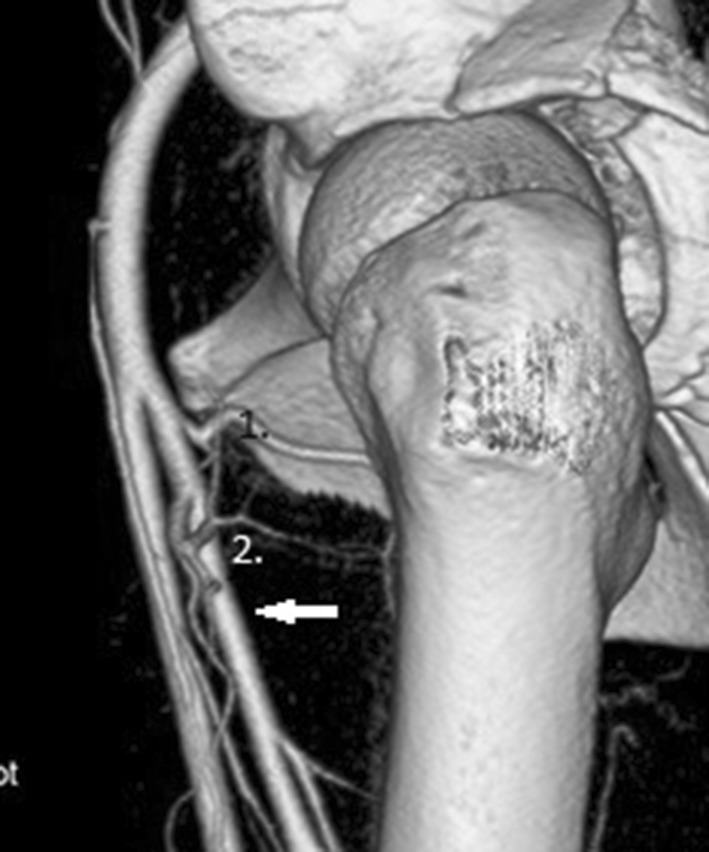
The volume rendering transformation of the angio CT examination of the truncus profundocircumflexus perfectus, MFCA (1), LFCA (2), deep femoral artery (arrow)

Many different types of division have been described. The most frequent one, truncus profundocircumflexus perfectus, named after Adachi [1] in which both the MFCA and LFCA originate from the deep femoral artery, was observed in 50% of patients (Fig. 1).

**Fig. 2:**
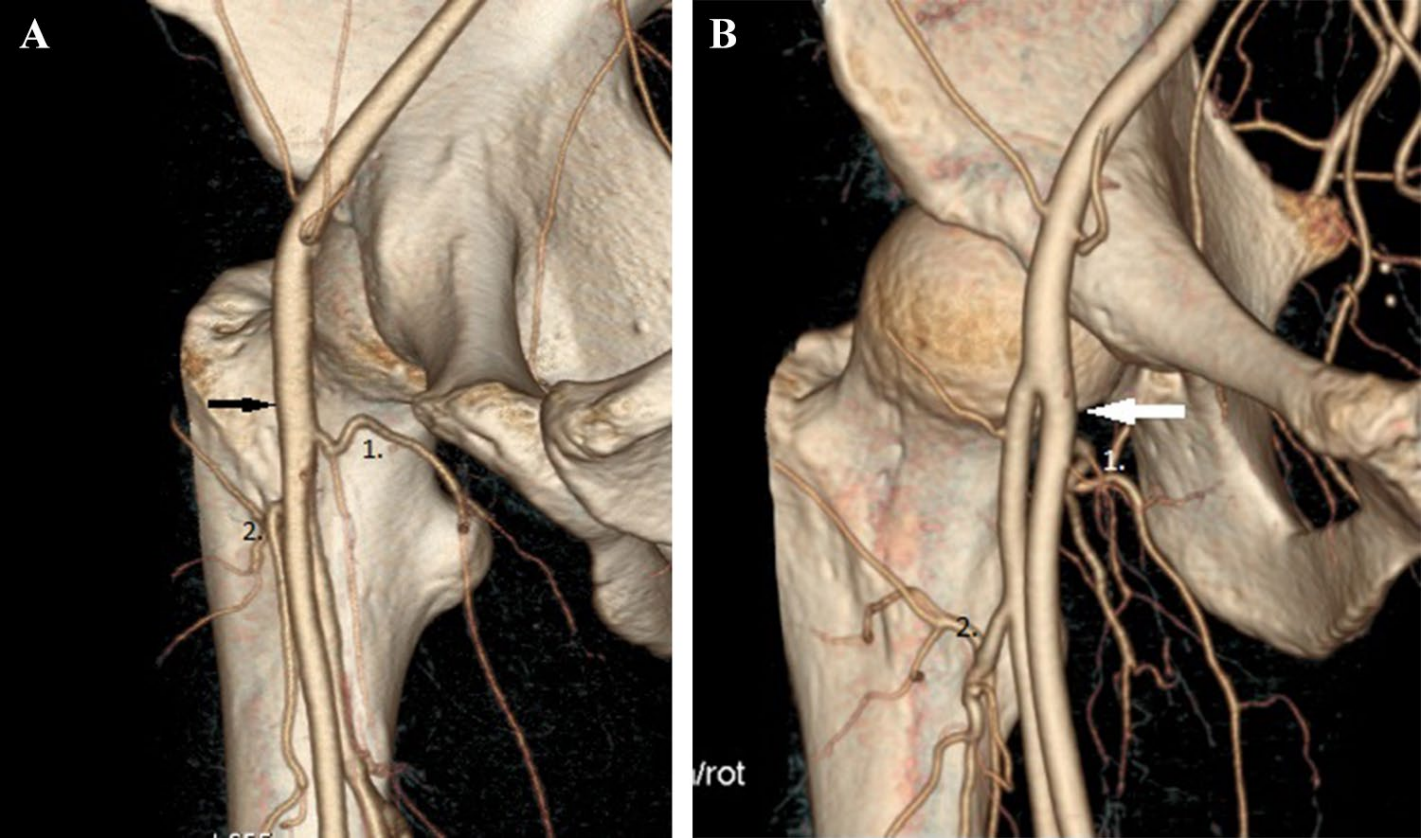
**a** Truncus profundocircumflexus lateralis type A, MFCA (1) originates from the common femoral artery (arrow), LFCA (2) originates from the deep femoral artery. **b** Truncus profundocircumflexus lateralis type B, MFCA(1) originates from the superficial femoral artery (arrow), LFCA (2) originates from the deep femoral artery

In 31% of hips, we found the truncus profundocircumflexus lateralis type of division. In that type, the MFCA originates from the common or superficial femoral artery and the LFCA from the deep femoral artery. In 28 of 31 hips, the MFCA originates from the common femoral artery (before the division into the deep femoral artery) (type A), and in 3 of 31 hips, the MFCA originates from superficial femoral artery (after the division into the deep femoral artery) (type B). These types are shown in Fig. 2a, b.

**Fig. 3:**
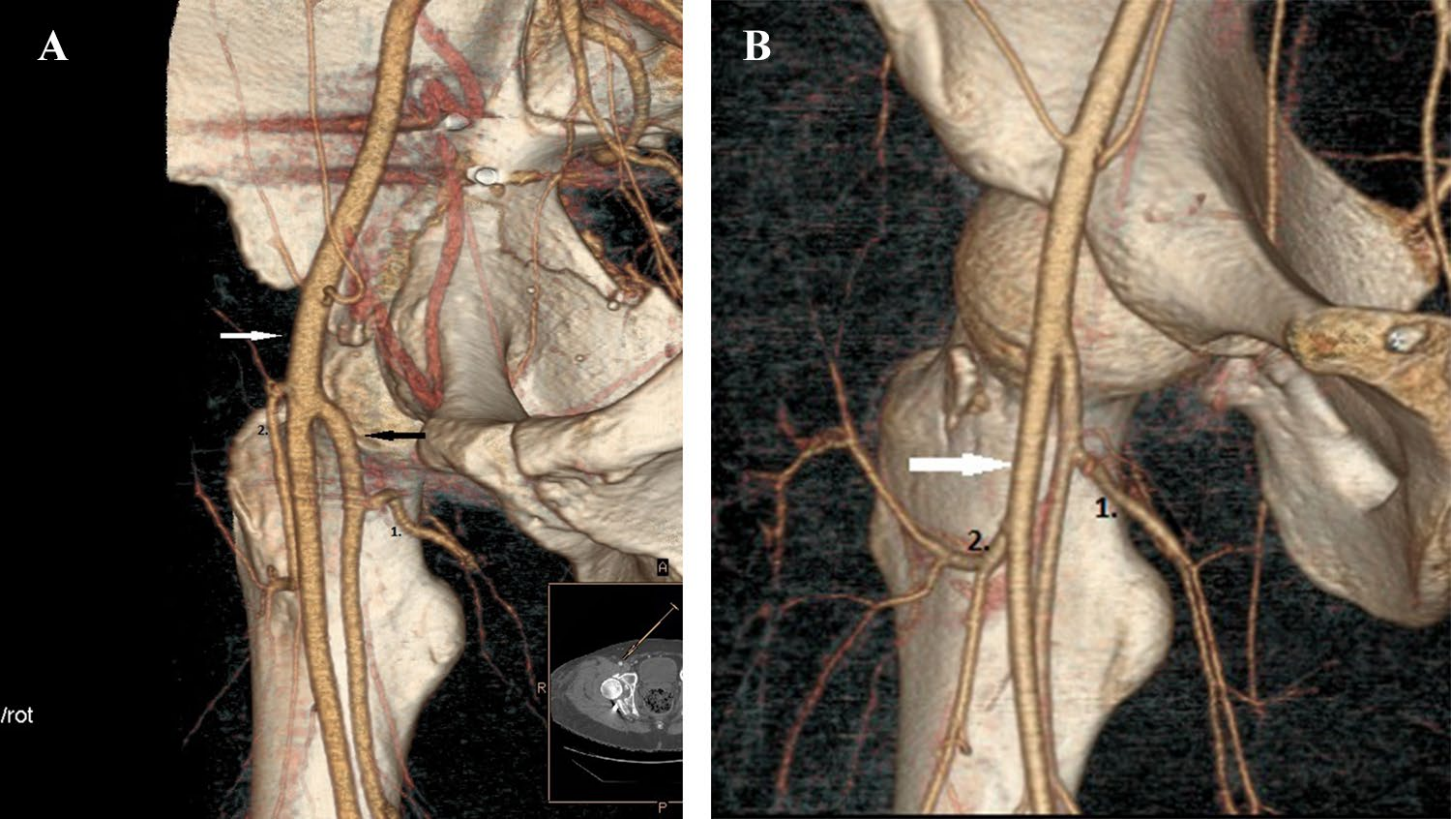
**a** Truncus profundocircumflexus medialis type A LFCA (2) originates from the common  femoral artery (white arrow), MFCA (1) from the deep femoral artery (black arrow). **b** Truncus profundocircumflexus medialis type B LFCA (2) originates from the superficial femoral artery (white arrow), MFCA (1) from the deep femoral artery

In 15%, we observed the truncus profundocircumflexus medialis type of division, where the MFCA originates from the deep femoral artery and the LFCA from the common or superficial  femoral artery. In 13 of 15 hips, the LFCA originates from the common femoral artery (type A), and in 2 of 15 hips, the LFCA originates from superficial femoral artery (type B). These types are shown in Fig. 3a, b.

**Fig. 4:**
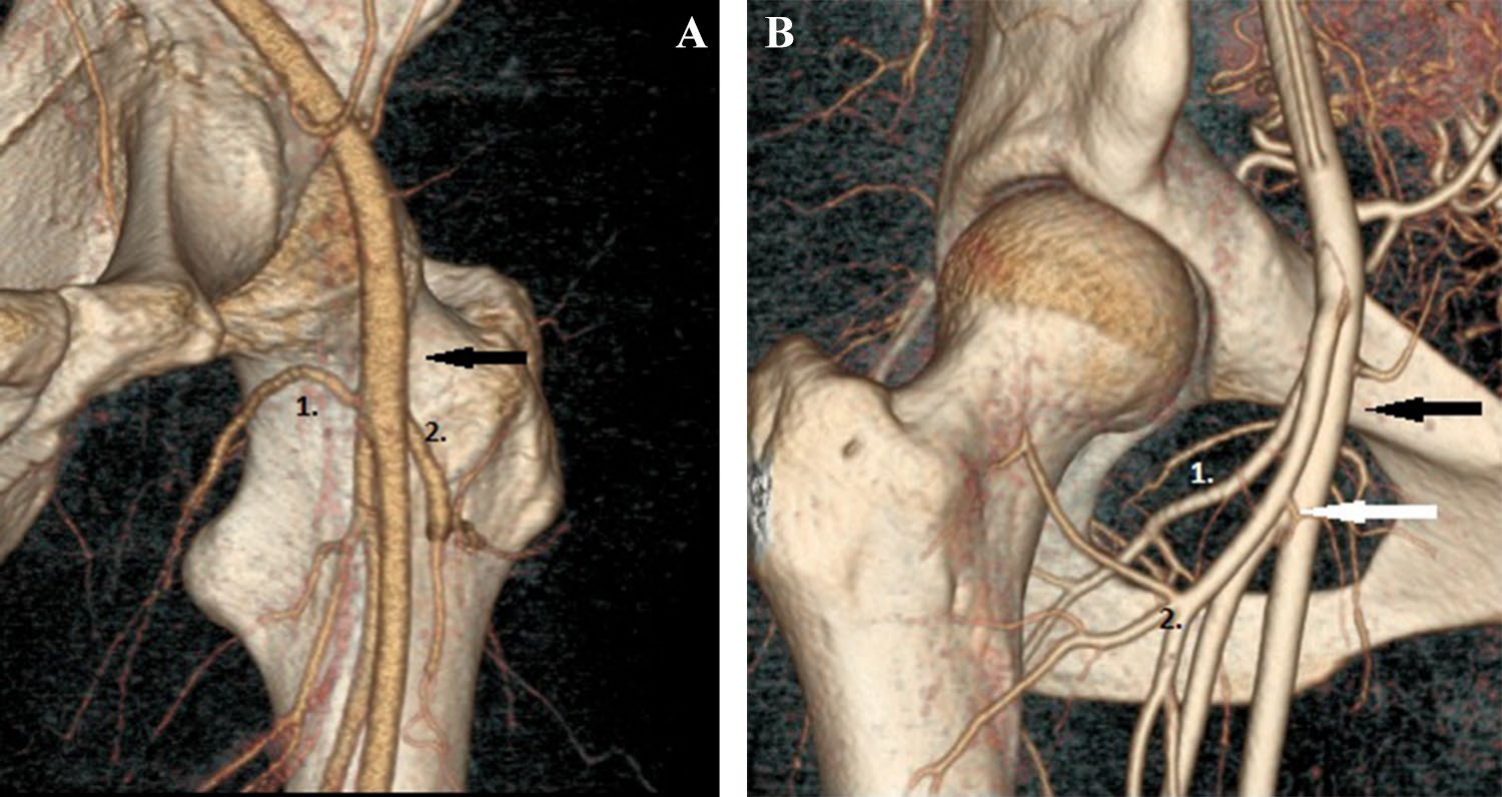
**a** MFCA (1) and LFCA (2) originate from common femoral artery (arrow). **b** MFCA (1) and LFCA (2) originate as one common trunk from the common femoral artery (black arrow) before the division (white arrow) into deep femoral artery and superficial femoral artery

**Fig. 5:**
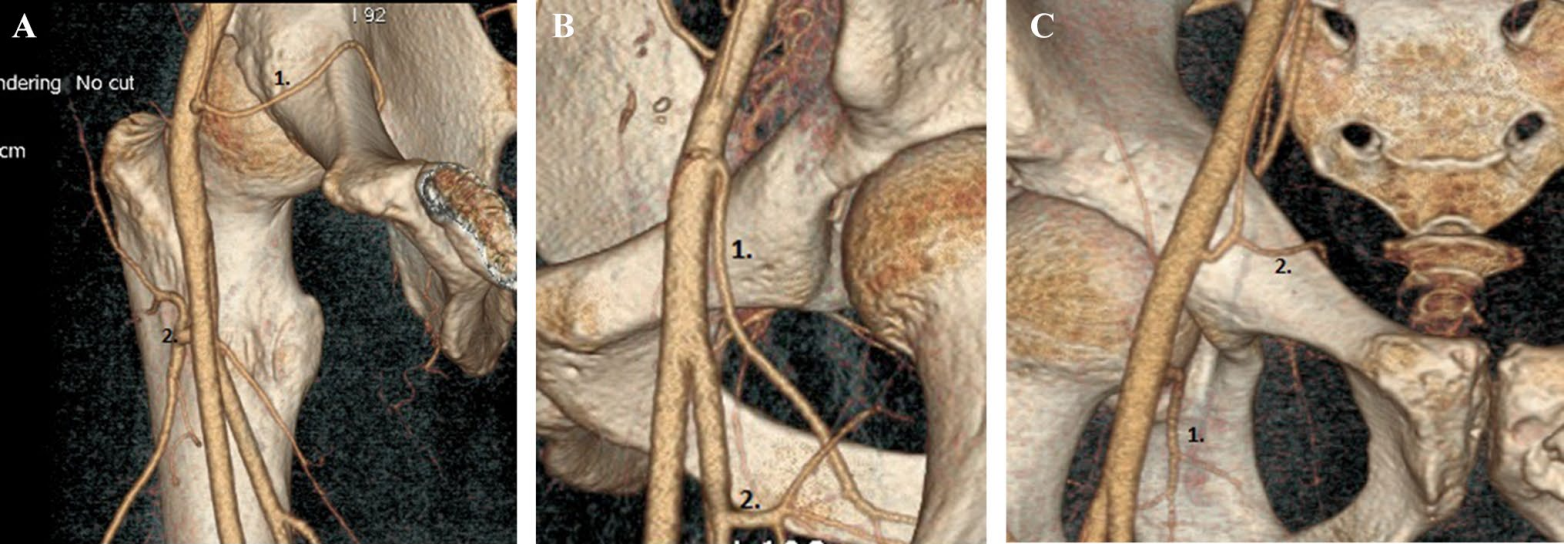
A case with no MFCA with an atypical obturator artery (1), LFCA (2) (**a**), in comparison with a case with a high level of division of the MFCA (1) directly from the common femoral artery located above the level of the pubic bone (**b**), the third case, with both arteries, represents an atypical obturator artery (2) and an MFCA (1) arising directly from the  common femoral artery (**c**)

In two cases, both the MFCA and LFCA originate from common femoral artery (Fig. 4a), and in another 1 case both the MFCA and LFCA originate as one trunk from common femoral artery (Fig. 4b). In 1 case no MFCA was visible (Fig. 5a). All types of division are shown in Table 1.

**Table 1:** All types of division in our material

Type of division	Incidence
Truncus profundocircumflexus perfectus MFCA and LFCA originate from deep femoral artery	50% (50/100)
Truncus profundocircumflexus lateralis LFCA originates from deep femoral artery Type A (MFCA originates from common femoral artery) Type B (MFCA originates from superficial femoral artery)	31% (31/100) 28/31 3/31
Truncus profundocircumflexus medialis MFCA originates from deep femoral artery Type A (LFCA originates from common femoral artery) Type B (LFCA originates from superficial femoral artery)	15% (15/100) 13/15 2/15
MFCA and LFCA directly from common femoral artery	2/100
MFCA and LFCA directly from common femoral artery as one trunk	1/100
No MFCA	1/100

By analyzing the pattern of origin of the MFCA from  common femoral artery, superficial femoral artery or deep femoral artery, we found that MFCA originates from the deep femoral artery in 65/100 patients, from the common femoral artery in 31/100 patients, from the superficial femoral artery in 3/100 patients, the origin was not found in one case.

We compared the type of division in the right hip region to that in the left hip region in the same individual and analyzed both hips in 73 patients. The same type of division on both sides was seen in 53.4% patients, whereas the rest of the patients 46.6% had different types of division on the right and left sides.

The level from which the MFCA arises frequently varies.

A high level of division was seen in the truncus profundocircumflexus lateralis type of division, where it was located a maximum of 52.5 mm above the bifurcation of the common femoral artery into the deep and superficial femoral artery. A low level of division was seen in the truncus profundocircumflexus medialis type of division and was located a maximum of 52 mm distally to the common femoral artery bifurcation.

The obturator artery has many variations in its topography. Normally, it arises from the iliac internal artery with other branches, like the gluteus superior artery and gluteus inferior artery. In its course through the obturator foramen, it anastomoses with the pubic branch of the inferior epigastric artery arising from the external iliac artery. Atypically, the pubic branch of the inferior epigastric artery is the dominant vessel of that anastomosis and the original obturator artery is atrophic or absent. In this atypical situation, the obturator artery arises from the inferior epigastric artery or directly from the external iliac artery. An anastomosis located around the pubic bone is called corona mortis because of a high risk of iatrogenic damage during several surgical and orthopedic procedures [10, 14].

In our study, a normal origin of the obturator artery from the internal iliac artery was observed in 67% of patients, and an atypical origin called corona mortis was observed in 33% of patients.

The incidence of an atypical division of the obturator artery on both sides was seen in 52% of patients. In 48% of patients it was seen only on one side (on the right in 9/16 patients and on the left in 7/16 patients) (Table 2).

**Table 2:** Variations in the atypical origin of the obturator artery

Anatomical variation	Incidence
*Corona mortis* anastomosis	33% (33/100)
*Corona mortis* bilaterally	52% (17/33)
*Corona mortis* unilaterally	48% (16/33)
*Corona mortis* unilaterally on right side	56% (9/16)
*Corona mortis* unilaterally on left side	44% (7/16)

As mentioned above, no mean trunk of the MFCA was found in one case.

In that case, the obturator artery originating atypically from the inferior epigastric artery was well contrasted. After its common origin with the inferior epigastric artery, the obturator artery took the course around the superior margin of the ramus superior of the pubic bone, then entered the obturator foramen and went toward the minor trochanter, where it anastomosed with the piriformis branch of the inferior gluteal artery.

The rare anastomosis with the piriformis branch that provides the blood supply to the femoral head may be somehow connected with the absence of the MFCA, the main vessel supplying the femoral head. A more distal origin of the common trunk of the epigastric inferior and obturator arteries than usual, distal to the inguinal ligament (in that case with no MFCA) can suggest that the main trunk of the MFCA took the course of the corona mortis during its development. To visualize that hypothesis, we present the case mentioned above in comparison with a case in which a high level of division of the MFCA from the common femoral artery is located near the pubic bone (Fig. 5a–c).

## Discussion

The most common type of division of the medial and lateral femoral circumflex arteries was the truncus profundocircumflexus perfectus, which was found in 50/100 patients.

By analyzing the pattern of origin of the MFCA, we found that the MFCA originated from the deep femoral artery in 65% of patients, from common femoral artery in 31% of patients, from superficial femoral artery in 3% of patients, it was not found in one case.

The same type of division on both sides was seen in 53% of patients.

We have compared our results with those of other studies. Tanyelli’s study [20] (100 hips, 50 patients) found the MFCA arising from the deep femoral artery in 81% of patients and from the femoral artery in 15% of patients; he found a double MFCA (one branch from the femoral artery and another from the deep femoral artery) in 4% of patients.

In a study by Adachi (1928) [1], the MFCA was found to originate from the deep femoral artery in 67.2% of patients and from the femoral artery in 14% of patients.

Another study by Lippert (1985) [11] showed the MFCA originating from the deep femoral artery in 58% of patients and the femoral artery in 18% of patients. In a study conducted by Siddharth (1985) [19], the MFCA originated from the deep femoral artery in 63% of patients and the femoral artery in 26% of patients, and the respective values in a study by Massound (1997) [13] were 81 and 6,4% of patients.

According to Al-Talalwah (2015) [3] MFCA the most frequently arose from deep femoral artery in 57%, secondly from common femoral artery in 39.3%, and less frequently from superficial femoral artery in 2.5%, LFCA in 0.6% and it was absent in 0.6%.

In the metaanalysis conducted by Tomaszewski (2016) [21] the prevalence pooled from 38 studies of MFCA originating from deep femoral artery was 64.6%, while from the common femoral artery was in 32.2%.

In our study, we found the MFCA originating from the deep femoral artery in 65% of patients, from the common femoral artery in 31% of patients and from superficial femoral artery in 3%.

The atypical origin of the obturator artery from the external iliac artery, called corona mortis, was described by many researchers. Adachi [1] found differences between European and Japanese populations (28.2 and 13.2%, respectively), in women 6.1% more often than in men. Lippert [11] and Bergmann [4] found the corona mortis in 20–30% of patients. In the study by Sanudo (2011) [17] the corona mortis was present in 31% of specimens, in 58.93% on both sides of the pelvis. Al Talalwah (2016) [2] performed a cadaveric study on the Austrian population, and the corona mortis variation was found in 12%. Rusu (2009) [15] defines corona mortis not only as arterial connection between the external iliac and obturator vascular systems, but also as a venous anastomosis between these systems. In that study “arterial” corona mortis was observed in 65% of hemipelvises, and “venous” anastomosis in 55% of specimens. In the study by Yiming [23] obturator artery arises from the external iliac artery in two cases out of ten. Several authors [5, 16, 18] described a rare arterial variation—a common arterial trunk which divides into the obturator artery, inferior epigastric artery and profunda femoris artery from which arises the medial femoral circumflex artery. Bilgic [5] suggests it is a result of the complicated embryologic development of the arteries of the lower limb.

According to Bergman’s Comprehensive Encyclopedia of Human Variation the frequency of the obturator artery which arises from the external iliac artery varies from 23.7–38.3%. It is found more often in females 33.2% than in males 28.0%. Depending on the author the asymetrical pattern on the left and right side varies from 9–57.4%. In literature the corona mortis is not clearly defined and distinguished from the obturator artery originating from the external iliac artery or iliopubic anastomosis [22].

In our study, the normal origin of the obturator artery from the internal iliac artery was observed in 67% of patients, and an atypical origin (called corona mortis) was observed in 33% of patients.

We found a rare anastomosis of the corona mortis with the piriformis branch of the inferior gluteal artery that provided the blood supply to the femoral head when the main trunk of the MFCA was absent. This anatomical variation can suggest that the main trunk of the MFCA took the course of the corona mortis during its development. We have not found such hypothesis in the literature.

The deep branch of the medial femoral artery and the piriformis branch of the inferior gluteal artery are the main arteries supplying the femoral head. Therefore, knowledge of their topography is very important during surgery in the hip region to prevent iatrogenic damage with an avascular necrosis of the femoral head [24]. The knowledge of the anatomy of corona mortis is essential in pelvic surgery (ilioinguinal approach to fractures of the acetabulum, pelvic osteotomies) to prevent bleeding which can be difficult to control in that anatomical location.

Although the resemblance of the high origin of the MFCA from the common femoral artery to the common trunk of the inferior epigastric and corona mortis is very intriguing, it needs more embryological studies to verify the hypothesis of similar development of those arteries.
